# Synaptic Properties and Plasticity Mechanisms of Invertebrate Tonic and Phasic Neurons

**DOI:** 10.3389/fphys.2020.611982

**Published:** 2020-12-16

**Authors:** Nicole A. Aponte-Santiago, J. Troy Littleton

**Affiliations:** ^1^The Picower Institute for Learning and Memory, Department of Biology and Department of Brain and Cognitive Sciences, Massachusetts Institute of Technology, Cambridge, MA, United States; ^2^Eli and Edythe Broad Center of Regeneration Medicine and Stem Cell Research, Department of Obstetrics, Gynecology and Reproductive Sciences, University of California, San Francisco, San Francisco, CA, United States

**Keywords:** Drosophila, synaptic transmission, synaptic plasticity, synapse, tonic, phasic, neuromuscular junction

## Abstract

Defining neuronal cell types and their associated biophysical and synaptic diversity has become an important goal in neuroscience as a mechanism to create comprehensive brain cell atlases in the post-genomic age. Beyond broad classification such as neurotransmitter expression, interneuron vs. pyramidal, sensory or motor, the field is still in the early stages of understanding closely related cell types. In both vertebrate and invertebrate nervous systems, one well-described distinction related to firing characteristics and synaptic release properties are tonic and phasic neuronal subtypes. In vertebrates, these classes were defined based on sustained firing responses during stimulation (tonic) vs. transient responses that rapidly adapt (phasic). In crustaceans, the distinction expanded to include synaptic release properties, with tonic motoneurons displaying sustained firing and weaker synapses that undergo short-term facilitation to maintain muscle contraction and posture. In contrast, phasic motoneurons with stronger synapses showed rapid depression and were recruited for short bursts during fast locomotion. Tonic and phasic motoneurons with similarities to those in crustaceans have been characterized in Drosophila, allowing the genetic toolkit associated with this model to be used for dissecting the unique properties and plasticity mechanisms for these neuronal subtypes. This review outlines general properties of invertebrate tonic and phasic motoneurons and highlights recent advances that characterize distinct synaptic and plasticity pathways associated with two closely related glutamatergic neuronal cell types that drive invertebrate locomotion.

## Introduction

With few exceptions, every cell in an animal has the same gene set encoded in their chromosomal DNA. Yet different cells navigate unique paths to differentiation and express only a subset of the individual genes that define what that cell becomes and how it works within the organism as a whole. There is no place where that diversity is on display more than in the nervous system. Thousands of individual cell types are found in the brain, each forming connections with many other neurons. This developmental feat gives rise to a biological machine that processes external stimuli and combines it with internal motivation states and prior experiences to guide ongoing behavior. Beyond the remarkable diversity of cell types, neurons can also rapidly alter the genes they express to guide modifications in their activity and structure that contribute to behavioral plasticity (Sheng and Greenberg, 1990; Sheng et al., 1990; Guan et al., 2005; Leslie and Nedivi, 2011; Crocker et al., 2016; Yap and Greenberg, 2018; Gray and Spiegel, 2019). How neurons create unique functional and structural identities and still allow flexible changes to occur during states of plasticity is a fundamental question in neuroscience. Indeed, severe neurodevelopmental and neurodegenerative diseases can occur when these processes are disrupted (Melom and Littleton, 2011; Mayford et al., 2012; Doll and Broadie, 2014; Nahmani and Turrigiano, 2014).

A goal for many in the field has been to unravel the genomic complexity of neurons and create comprehensive brain cell atlases (Ecker et al., 2017; Zeng and Sanes, 2017). Can one decipher which of the thousands of genes available to a neuron are ultimately expressed? More importantly, which gene combinations drive emergence of unique functional and structural properties for each neuronal class? In addition, what subset of these differentially expressed genes enable the neuron to form preferential connections to synaptic partners from a large cohort of potential choices? Deciphering these fundamental questions in neuronal diversity and connectivity will empower broad efforts in neuroscience to understand how the brain is built and how it functions. With modern molecular techniques in cell biology, single cell RNA profiling experiments can be performed to determine which of the genes encoded in an animal’s genome are expressed, along with relative mRNA abundance within individual neurons (Belgard et al., 2011; Darmanis et al., 2015; Cadwell et al., 2016; Fuzik et al., 2016; Lake et al., 2016; Poulin et al., 2016; Chen et al., 2017; Brunet Avalos et al., 2019; Ortiz et al., 2020). However, even with a known transcriptome, the challenge of understanding what specific gene expression signatures mean for any class of neurons remains. More divergent cell types are likely to display greater diversity in their transcriptomes compared to closely related ones (Arendt et al., 2016). Given the complexity of neuronal diversity, an attractive approach is to simplify the question – to discover how distinct transcriptional programs generate diversity in neuronal function and connectivity at the level of closely related neuronal subgroups that show modest differences in their properties. Invertebrate tonic and phasic motoneurons represent an interesting and highly related subgroup of glutamatergic neurons to begin deciphering the molecular underpinnings of neuronal and synaptic diversity. Here we describe recent advances in understanding the diversity of these neuronal subclasses and look toward the future at potential approaches to generate a more detailed view of the key molecular engines that drive biophysical and synaptic heterogeneity.

## Overview of Tonic and Phasic Neuronal Subtypes

Synapses are key sites where information is transferred between neurons. Changes to their structure or function can alter local information flow or circuit activity as a general mechanism for behavioral plasticity. Indeed, synaptic competition during assembly of neural circuits has emerged as an important component of brain development. Although activity-dependent synaptic competition is widely studied, how plasticity of inputs from distinct neuronal classes are regulated and the role of post-synaptic cells in this process is still being elucidated. Much of the work in this area has focused on the interplay between excitatory and inhibitory inputs that control overall output of a circuit, along with the neurodevelopmental disorders that occur when the process is disrupted (Davis and Bezprozvanny, 2001; Gogolla et al., 2009; Yizhar et al., 2011; Zoghbi and Bear, 2012; Davis, 2013; Deisseroth, 2014; Nelson and Valakh, 2015; Lee et al., 2017). Among interactions of excitatory inputs, tonic and phasic neurons co-innervate many post-synaptic targets and provide distinct patterns of excitatory drive.

Tonic and phasic neuronal subtypes were initially defined in vertebrates based on distinct excitability properties, with tonic neurons firing in a sustained manner and phasic neurons displaying burst properties with rapid adaptation. Studies of invertebrate locomotion revealed motoneuron subclasses with similar differences in excitability properties. Although initial studies of phasic and tonic motoneurons where described in amphibians (Kuffler and Vaughan Williams, 1953a,b), studies in crayfish provided insights into how unique motoneuron output characteristics can drive animal locomotion (Atwood, 2008). Indeed, crustacean muscles emerged as an early model for this type of co-innervation. Two distinct inputs important for locomotion were characterized, including a “tonic” slowly contracting, sustained, fatigue-resistant cycle and a quick, twitch-like, non-sustained “phasic” cycle (Atwood, 1963, 2008; Takeda and Kennedy, 1964, 1965; Bradacs et al., 1997; Msghina et al., 1998; Millar and Atwood, 2004). This system employs two unique motoneuron subtypes with distinct properties that co-innervate some muscles and individually innervate others (Lnenicka, 2020). Although synaptic inputs are likely distinct for tonic and phasic motoneurons based on local central nervous system (CNS) circuity, direct current injection into these neuronal subtypes can trigger their unique firing properties (Choi et al., 2004; Schaefer et al., 2010), suggesting unique excitability differences that are genetically encoded. For individual targets, phasic neurons typically innervate larger muscles used for escape behaviors, while tonic neurons project to thinner muscles required to maintain spontaneous activity for locomotion and posture (Atwood, 2008). Studies in muscles of the cat limb described a behavioral parallel to some of the crustacean work, with tonic and phasic outputs implicated in posture and walking, respectively (Dum and Kennedy, 1980; McDonagh et al., 1980; Zengel et al., 1985).

**FIGURE 1:**
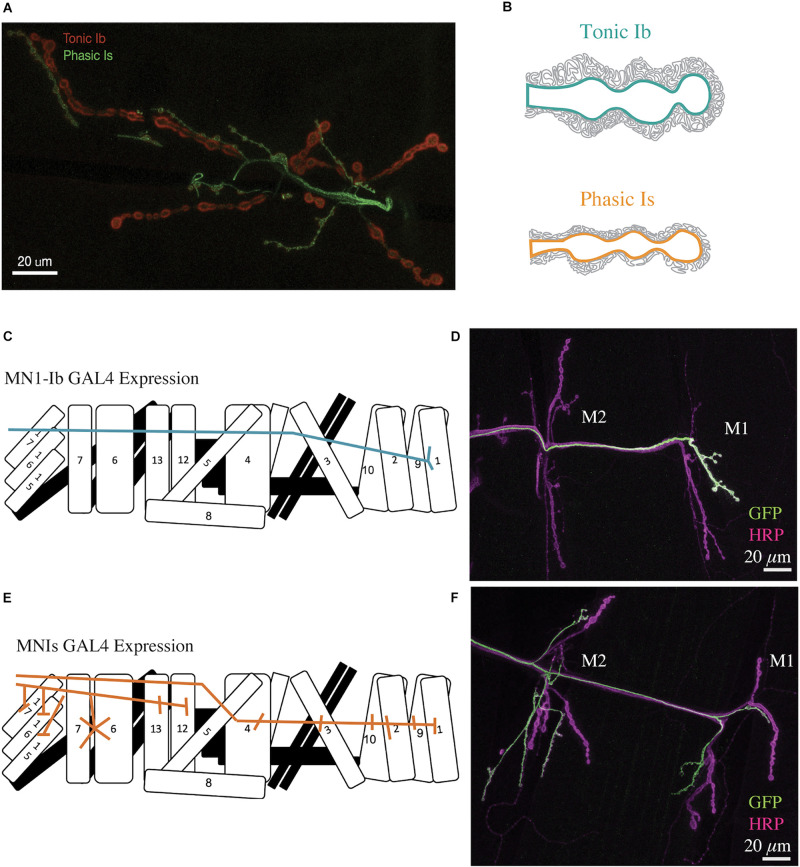
Structure of Drosophila tonic Ib and phasic Is motoneuron terminals. **(A)** Representative confocal image of a Drosophila 3rd instar larval muscle 6/7 NMJ. Immunolabeling for the PSD protein Dlg is shown in red. The phasic Is neuron is labeled green with a Is-GAL4 specific line driving UAS-GFP. Dlg is found throughout the muscle subsynaptic reticulum (SSR) and is more prevalent surrounding the bigger Ib boutons (red). **(B)** Diagram depicting muscle SSR invaginations around tonic Ib (teal) and phasic Is (orange) boutons. **(C)** Diagram of MN1-Ib motoneuron innervation of only muscle 1 in a larval abdominal hemi-segment. **(D)** Immunostaining for anti-GFP (green) to label MN1-Ib and HRP (magenta) to label all axons in a MN1-Ib GAL4; UAS-CD8-GFP 3rd instar larva. The location of muscles M1 and M2 are indicated. Scale bar = 20 μm. **(E)** Diagram of MNISN-Is and MNSNb/d-Is innervation of muscles in a larval abdominal hemi-segment. **(F)** Immunostaining for anti-GFP (green) to label MNIs and HRP (magenta) to label all axons in a MNIs GAL4; UAS-CD8-GFP 3rd instar larva. The location of muscles M1 and M2 are indicated. Panels **(C–F)** are modified from Aponte-Santiago et al. (2020).

Although several differences in crustacean and Drosophila neuromuscular junctions (NMJs) have been described (Lnenicka and Keshishian, 2000; Millar and Atwood, 2004; Kohsaka et al., 2017; Lnenicka, 2020), the later has become a popular system for characterizing the genetic underpinnings of distinct tonic and phasic motoneuron properties. The Drosophila larval motor system has a stereotypical segmental development with each abdominal half-segment containing 30 muscles innervated by ∼36 identifiable motoneurons (Johansen et al., 1989; Sink and Whitington, 1991b; Atwood et al., 1993; Hoang and Chiba, 2001; Harris and Littleton, 2015; Clark et al., 2018; Arzan Zarin and Labrador, 2019). These motoneurons form four unique subclasses defined by their functional properties, presynaptic bouton structure and innervation pattern (Jan and Jan, 1976; Johansen et al., 1989; Atwood et al., 1993; Lnenicka and Keshishian, 2000; Hoang and Chiba, 2001). Type I motoneurons are glutamatergic and subdivided into the Ib class with “big” boutons (3–6 μm in diameter) and the Is class that has “small” boutons (2–4 μm in diameter) as shown in Figure 1A. The Ib and Is neuronal subtypes have distinct morphological and electrophysiological properties that led to their classification as tonic (Ib) or phasic (Is) based on some similarities to crustaceans (Johansen et al., 1989; Atwood et al., 1993; Jia et al., 1993; Kurdyak et al., 1994; Msghina et al., 1998; Lnenicka and Keshishian, 2000; Hoang and Chiba, 2001; Lu et al., 2016; Newman et al., 2017; Aponte-Santiago et al., 2020). Around 30 Ib motoneurons individually innervate each of the 30 muscles in a hemi-segment during late embryogenesis, while three Is neurons per hemi-segment innervate subsets of muscles to coordinate contraction of specific subgroups. Innervation of abdominal muscles by Is motoneurons typically follows innervation by their Ib counterparts, with some muscles occasionally lacking Is input altogether (Ashley et al., 2019; Aponte-Santiago et al., 2020). The terminal axons of Ib and Is motoneurons continue to grow over the muscle surface during the subsequent 6 days of larval development to eventually form ∼10 to 100 individual synaptic boutons depending on size of each specific muscle (Zito et al., 1999). Each bouton contains from ∼5 to 40 individual release sites known as active zones (AZs) that have a centrally located electron-dense T-bar that clusters synaptic vesicles (SVs). The remaining two classes of motoneurons in Drosophila larvae are neuromodulatory. Type II neurons contain only dense core vesicles (DCVs) and release the biogenic amine octopamine (Monastirioti et al., 1995; Stocker et al., 2018). A single type III peptidergic neuron innervates muscle 12 and releases insulin-like neuropeptide (Gorczyca et al., 1993). The well-characterized organization of the larval motor system, together with genetic approaches available in Drosophila, have made the system an attractive model to dissect functional and structural diversity of tonic and phasic motoneurons.

Although we focus our discussion on the motor system, it is worth noting that neurons with tonic and phasic properties have been described in other brain regions. In mammalian prefrontal cortex (PFC), tonic and phasic dopaminergic neurons contribute to behavioral flexibility associated with task switching vs. maintaining a learned behavior (Seamans et al., 1998; Durstewitz et al., 2000; Durstewitz and Seamans, 2002, 2008; Floresco and Magyar, 2006; Stefani and Moghaddam, 2006; St Onge et al., 2011, 2012; Puig and Miller, 2012, 2015). Tonic patterns of stimulation from dopamine neurons in the ventral tegmental area (VTA) cause mice to maintain a learned behavior. In contrast, reward produces phasic increases in activity of dopaminergic VTA-PFC fibers, resulting in changes to previously learned associations (Ellwood et al., 2017). In addition, tonic and phasic inhibition interact to maintain homeostasis in the mammalian brain. GABA_A_ receptors are located in both synaptic and extrasynaptic membranes in the brain and represent the primary receptors for inhibition. Synaptic GABA_A_ receptors mediate phasic inhibition, while extrasynaptic GABA_A_ receptors mediate tonic inhibition. Dysfunction in tonic or phasic inhibition has been associated with epilepsy, depression, and anxiety (Fritschy, 2008; Macdonald et al., 2010; Brickley and Mody, 2012; Hines et al., 2012). As such, tonic and phasic properties represent a common theme for several neuronal subclasses.

## Structure and Function of Invertebrate Tonic and Phasic Motoneurons

Studies in crustaceans and Drosophila have established several key differences at both the functional and structural level for tonic and phasic motoneurons (Table 1). At the behavioral level, recruitment of tonic Ib motoneurons during larval crawling results in a larger rise in muscle Ca^2+^ and represents the primary driver for contraction (Newman et al., 2017). In contrast, individual Is motoneurons innervate subsets of muscles and are predicted to coordinate contraction for specific locomotor tasks. Elimination of larval Is motoneurons using GAL4-mediated cell ablation does not cause lethality (Aponte-Santiago and Littleton, unpublished data), but detailed studies of the overall consequences on larval locomotion have not been performed. Connectomic studies of the larval motor system indicate Ib and Is motoneurons have both unique and shared pre-motor inputs and may receive temporally distinct inhibitory and excitatory drive during specific locomotor tasks (Zarin et al., 2019). More functional studies are required to understand how the two distinct motoneuron types cooperate to control larval locomotion.

**TABLE 1:** Differences in neuronal and synaptic properties of Drosophila tonic Ib and phasic Is motoneurons.

**Properties**	**Tonic (Ib)**	**Phasic (Is)**
Probability of release	Low P_r_	High P_r_
Short-term plasticity	Facilitation	Depression
Synaptic area and SSR	Larger	Smaller
Active zone number	Higher	Lower
Homeostatic plasticity	Chronic	Acute
Spiking threshold	Lower	Higher
Postsynaptic target innervation	Single muscle	Multiple muscles
Baseline [Ca2^+^]	Higher	Lower
Ca2^+^ sensitivity of release	Lower	Higher
AZ development	Slower	Faster
Mitochondrial and SV content	Higher	Lower
Role in locomotion	Posture, sustained contractions	Coordination of muscle groups

*Similar to other invertebrate tonic and phasic motoneurons, Drosophila tonic and phasic neurons show distinct innervation patterns, synaptic properties and membrane biophysics.*

Similar to studies in crustaceans, tonic and phasic motoneurons in Drosophila have substantially different membrane excitability profiles (Park et al., 2002; Choi et al., 2004; Worrell and Levine, 2008; Schaefer et al., 2010; Xing and Wu, 2018b). Patch-clamp recordings from identified Ib and Is larval motoneurons revealed that the phasic Is subtype requires more current injection to drive spiking. In addition, Is motoneurons display a more hyperpolarized resting membrane potential, larger input resistance, a longer delay to first spike following current injection, and fewer overall spikes during a burst. Similar observations have been made with optical approaches to image Ca^2+^ influx, where Ib motoneurons are engaged earlier during locomotion (Newman et al., 2017). The differences in spike timing in the two populations have been mapped to the *I*_A_ K^+^ channel current encoded by the *Shal* gene, which is predicted to mediate earlier recruitment of low threshold Ib motoneurons before high threshold Is neurons are engaged (Choi et al., 2004; Schaefer et al., 2010). Distinct excitatory and inhibitory input onto Is and Ib motoneurons by CNS interneurons also regulates the recruitment of each subclass between and within abdominal hemi-segments (Heckscher et al., 2015; Fushiki et al., 2016; Zwart et al., 2016; Zarin et al., 2019). Therefore, a combination of both intrinsic properties and local interneuron circuitry contribute to spiking differences between Ib and Is motoneurons during locomotion.

**FIGURE 2:**
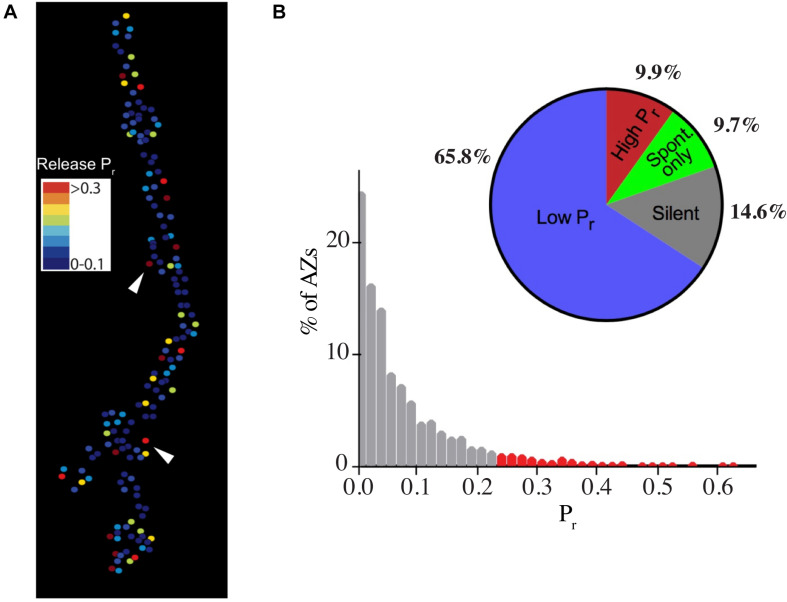
Heterogeneity in synaptic transmission strength of individual AZs at Ib motoneuron terminals. **(A)** Heat map for evoked AZ *P*_*r*_ at Ib NMJs at 3rd instar muscle 4 determined by optical quantal imaging with post-synaptic myristoylated GCaMP6s. Stronger AZs are shown in red with weaker AZs displayed in the colder blue colors. Arrowheads denote several high *P*_*r*_ AZs. **(B)** Histogram of AZ *P*_*r*_ distribution for 0.3 Hz stimulation for 5 min for Ib motoneurons. AZs classified as high *P*_*r*_ (>2 standard deviations above the mean) are shown in red. The pie chart shows the percentage of overall AZs from multiple NMJ optical imaging sessions that represent low *P*_*r*_ (65.8%), high *P*_*r*_ (9.9%), spontaneous-only (9.7%), or silent (14.6%) AZs for the Ib motoneuron population innervating muscle 4. Note the pie chart colors are unique and do not reflect the *P*_*r*_ heatmap. Panels **(A,B)** are modified from Akbergenova et al. (2018).

In both Drosophila and crustaceans, phasic motoneurons have stronger synapses that release more neurotransmitter than their tonic counterparts. This is especially apparent in crustaceans where quantal content, defined as the number of SVs released per action potential, can be 100- to 1000-fold greater at phasic synapses (Msghina et al., 1999). The differences in quantal content in Drosophila are more modest, with phasic Is synapses releasing two- to three-fold more SVs than tonic Ib motoneurons (Lu et al., 2016; Newman et al., 2017; Genç and Davis, 2019; Aponte-Santiago et al., 2020; Karunanithi et al., 2020a; Wang et al., 2020). These differences in output have been mapped to changes in SV release probability (*P*_*r*_–the likelihood a SV fuses following an action potential) at single AZs, indicating individual release sites have on average higher *P*_*r*_ at phasic Is terminals (Lu et al., 2016; Newman et al., 2017). Although Is AZs have a higher average *P*_*r*_, optical quantal imaging of single AZ release properties has revealed a wide heterogeneity in evoked *P*_*r*_ across the 100s of AZs formed by both motoneuron types (Figures 2A,B). Synaptic strength ranges from silent and low *P*_*r*_ sites (<0.2) to a smaller fraction (∼10%) of high *P*_*r*_ AZs (0.2–0.7) depending on extracellular [Ca^2+^] (Peled and Isacoff, 2011; Melom et al., 2013; Peled et al., 2014; Newman et al., 2017; Akbergenova et al., 2018). Much of this variability in *P*_*r*_ has been mapped to increased accumulation of presynaptic Ca^2+^ channels and late AZ scaffolding proteins like Bruchpilot (BRP) at high *P*_*r*_ AZs in Ib terminals (Akbergenova et al., 2018). Overall, the distribution of *P*_*r*_ values shift in Is motoneurons such that they contain more high *P*_*r*_ AZs compared to their Ib counterparts.

Although phasic AZs release more neurotransmitter compared to tonic sites, the underlying molecular mechanisms at play are still being defined. There is evidence suggesting both functional and structural mechanisms are involved. Initial studies in crayfish found that intra-terminal Ca^2+^ measured with the Fura-2 Ca^2+^ indicator following nerve stimulation was five-fold higher at phasic terminals, suggesting an increase in Ca^2+^ influx could drive more SV fusion (Msghina et al., 1999). However, normalization of terminal Ca^2+^ levels to its removal rate and the number of individual AZs in each terminal led to a model where overall Ca^2+^ available to release sites would be relatively similar at tonic and phasic synapses. As such, differences in Ca^2+^ influx alone seemed unlikely to account for the 100- to 1000-fold increase in *P*_*r*_ at phasic terminals. Likewise, increases in the number of docked SVs and a larger readily releasable pool (RRP) were also ruled out as potential mechanisms. Indeed, tonic synapses were found to have a larger pool of docked SVs by EM (11 vs. 4) and a bigger RRP pool measured by electrophysiology (130 vs. 60) compared to phasic synapses (Millar et al., 2002). However, phasic synapses released ∼30% of their RRP compared to only ∼0.02% for tonic synapses. This observation suggests the underlying mechanism in crayfish is likely related to differences in the Ca^2+^ sensitivity of release, with phasic terminals requiring less Ca^2+^ influx to trigger SV release. What differences or modifications to the release machinery gives rise to such a dramatic increase (1000-fold) in *P*_*r*_ are currently unknown. Defining these mechanisms would be highly informative and potentially suggest molecular changes that might be harnessed for other forms of presynaptic plasticity throughout the nervous system (Atwood and Karunanithi, 2002).

Work in Drosophila suggests similar mechanisms may trigger the more modest enhancements in release observed at phasic Is terminals. As observed in crustaceans, measurements of presynaptic Ca^2+^ rise following single action potentials demonstrate higher levels in phasic synaptic boutons compared to tonic terminals (He et al., 2009; Lu et al., 2016). Again, differences in AZ number per bouton and the smaller size of phasic terminals have led to models that the overall Ca^2+^ available to AZs may be similar at Ib and Is synapses. Characterization of single AZ Ca^2+^ dynamics vs. the more global bouton-level changes measured to date will be required to determine if AZ Ca^2+^ influx contributes to *P*_*r*_ differences. Like crayfish, phasic terminals in Drosophila also display striking differences in the Ca^2+^ sensitivity of release compared to their tonic counterparts (Genç and Davis, 2019). This difference results in elevated release rates in low Ca^2+^ conditions at phasic synapses, but similar output between tonic and phasic terminals in high extracellular Ca^2+^ when release saturates (Lu et al., 2016; Newman et al., 2017; Genç and Davis, 2019; Karunanithi et al., 2020a). Whether changes in the abundance or properties of the Ca^2+^ sensor Synaptotagmin or other components of the SV fusion machinery at Is vs. Ib terminals participate in these differences in Ca^2+^ sensitivity is unknown. One intriguing finding is the observation that resting baseline Ca^2+^ levels appear to be lower at phasic Is terminals compared to Ib (Xing and Wu, 2018a). As such, a greater driving force for Ca^2+^ entry at phasic terminals could also contribute to the enhanced *P*_*r*_. The TRP channel Inactive has been shown to play a key role in setting resting Ca^2+^ levels at Drosophila NMJs, and it will be interesting to determine if differences in the amount or activity of this channel contribute to alterations in resting Ca^2+^ at Ib vs. Is terminals (Wong et al., 2014).

Beyond differences in the Ca^2+^ sensitivity of release and resting Ca^2+^ levels, several morphological features may also contribute to stronger synapses in phasic motoneurons. Phasic synapses have slightly larger individual AZ dense bars that could harbor up to 30% more Ca^2+^ channels in crayfish (King et al., 1996). Crayfish phasic terminals also display a larger proportion of multiple dense bars per AZ compared to those of tonic motoneurons. Likewise, phasic AZs rarely lack a presynaptic dense bar (2%), while tonic AZs have a higher proportion of synapses (12%) lacking this key structure that regulates SV docking and Ca^2+^ channel clustering (King et al., 1996). BRP is a key component of the electron dense T-bar structure that resides at the center of Drosophila AZs (Kittel et al., 2006; Wagh et al., 2006; Fouquet et al., 2009). The AZ levels of BRP increase during development and correlate with *P*_*r*_ at this synapse (Fouquet et al., 2009; Melom et al., 2013; Peled et al., 2014; Akbergenova et al., 2018). Indeed, the maturation state of AZs has been demonstrated to be a key factor in *P*_*r*_ heterogeneity at Drosophila NMJs (Akbergenova et al., 2018). Newly formed AZs have very low *P*_*r*_ and can take several days to mature into a high *P*_*r*_ state. Interestingly, Drosophila phasic Is synapses develop faster than their tonic Ib counterparts (Aponte-Santiago et al., 2020), suggesting a more rapid AZ maturation process at Is synapses may also contribute to their higher *P*_*r*_. Whether differences in synapse maturation at Is terminals reflects enhanced axonal transport of AZ cargo, distinct AZ maturation pathways, or activity-dependent steps triggered by the post-synaptic muscle requires further investigation. Finally, SV size is also different between Ib and Is motoneurons, with Is terminals containing ∼18% larger SVs (Karunanithi et al., 2002). This results in a ∼50% increase in spontaneous mini amplitude from SVs released by Is terminals when recorded with macropatch electrodes from isolated Is boutons. As such, phasic Is terminals have a higher average *P*_*r*_ per AZ that results in more SVs released over the entire axonal arbor, with each SV containing more neurotransmitter than those from tonic terminals.

Other morphological features that distinguish phasic and tonic terminals include the bigger boutons observed in Ib motoneurons that have increased mitochondrial and SV number compared to Is (Atwood et al., 1993; Jia et al., 1993). This difference, along with the activity of the plasma membrane Ca^2+^ ATPase (PMCA), appears to alter Ca^2+^ buffering and ATP production between the two terminal types. Indeed, PMCA plays a key role in Ca^2+^ extrusion at Ib terminals, with lower activity at Is synapses (Lnenicka et al., 2006; He et al., 2009). This decreased PMCA activity results in a slower rate of Ca^2+^ extrusion from phasic terminals during longer stimulation trains (He et al., 2009; Xing and Wu, 2018b). This is consistent with the higher firing frequency and longer burst duration of Ib motoneurons during fictive crawling in semi-intact larval preparations (40–60 Hz in Ib vs. 10–20 Hz in type Is) (Cattaert and Birman, 2001; Chouhan et al., 2010; Newman et al., 2017). The elevated firing activity of Ib neurons during locomotion is likely to require more robust Ca^2+^ clearance mechanisms than Is neurons to prevent intracellular Ca^2+^ buildup. Ib motoneurons also display higher ATP metabolism and a larger pool of synaptic mitochondria vs. Is terminals (Atwood et al., 1993; Jia et al., 1993; Xing and Wu, 2018b). Similar differences have been observed in crayfish tonic and phasic motoneurons (Bradacs et al., 1997; Nguyen et al., 1997; Msghina et al., 1998, 1999). Together with their lower AZ *P*_*r*_, these properties are predicted to help maintain higher firing rates and persistent synaptic activity for tonic motoneurons during sustained muscle contraction cycles.

In addition to bouton size, another morphological distinction between Ib and Is motoneurons in Drosophila is related to how the post-synaptic muscle membrane is organized around individual NMJs. Most larval muscles are co-innervated by a Ib and Is motoneuron. The axons of the Ib and Is motoneurons grow into the muscle as they expand during development. The invagination of the boutons into the muscle is highlighted by an expansive subsynaptic reticulum (SSR) with large infoldings of the muscle post-synaptic membrane that develop around the boutons over the course of larval development (Figure 1B). Mature Ib boutons have a far more expansive SSR than their Is counterparts (Johansen et al., 1989; Lnenicka and Keshishian, 2000). Disc large (DLG) is a well-known post-synaptic scaffolding protein that is more abundant in the SSR surrounding Ib terminals (Figure 1A). DLG is a member of the membrane-associated guanylate kinase (MAGUK) family. Similar MAGUK proteins like PSD-95 have roles in the organization of ionotropic glutamate receptors at mammalian post-synaptic densities (PSDs) (Elias and Nicoll, 2007; Sheng and Kim, 2011; Zheng et al., 2011). In Drosophila, DLG recruits several other key PSD proteins via its PDZ-binding domains, including the cell adhesion protein Fasciclin II (FasII), the Shaker K^+^ channel and the post-synaptic t-SNARE Gtaxin (Gtx) that controls SSR development (Budnik et al., 1996; Thomas et al., 1997; Zito et al., 1997; Chen and Featherstone, 2005; Gorczyca et al., 2007). The differential organization of the SSR around Ib and Is boutons indicates the muscle is capable of distinguishing between the two inputs and forming distinct post-synaptic specializations. This hypothesis is supported by the observation that glutamate receptor subtypes are differentially expressed at PSDs opposing the two inputs, with Ib synapses enriched for the GluRIIA subtype and Is terminals containing more of the GluRIIB subtype (Petersen et al., 1997; Marrus et al., 2004a; Rasse et al., 2005; Schmid et al., 2008; Aponte-Santiago et al., 2020). Drosophila glutamate receptors form tetramers with three shared subunits and a 4th subunit of either GluRIIA or GluRIIB (Schuster et al., 1991; Petersen et al., 1997; Marrus et al., 2004b; Featherstone et al., 2005; Qin et al., 2005). Whether the differential organization of the SSR around Ib and Is terminals is due to changes in the overall output of the two synaptic types or secondary to genetically hard-wired synaptic determinants is currently unknown. The observation that disrupting synaptic transmission in Drosophila Ib neurons reduces SSR development (Aponte-Santiago et al., 2020) and that morphology of crayfish phasic terminals can be driven to a more tonic appearance by long-term stimulation (Lnenicka et al., 1991, 1986; Nguyen and Atwood, 1994) suggest activity is likely to contribute to the differential post-synaptic development in both systems.

## Distinct Synaptic Plasticity Mechanisms for Tonic and Phasic Motoneurons

Neuronal plasticity occurs through multiple mechanisms across invertebrate and vertebrate species and is often associated with changes in synaptic strength or synapse number (Destexhe and Marder, 2004; Holtmaat and Svoboda, 2009; Mayford et al., 2012; Harris and Littleton, 2015). For example, changes in synapse morphology and number contribute to non-associative learning in Aplysia (Bailey and Chen, 1988, 1991, 1983; Kim et al., 2003; Mayford et al., 2012; Bailey et al., 2015). Similar experience-dependent plasticity mechanisms are also found in mammals (Foeller and Feldman, 2004). Changes in sensitivity to sensory input during vertebrate brain development has been well documented during temporal windows known as critical periods that result in widespread physiological and morphological changes. Hubel and Wiesel pioneered such studies in the cat visual system, establishing the concept of activity-dependence for a type of structural plasticity termed ocular dominance plasticity (ODP) (Wiesel and Hubel, 1965, 1963; Hubel and Wiesel, 1970). With monocular deprivation of one eye during critical periods, active axons from the open eye outcompete inactive axons to take over synaptic space in visual cortex (Hooks and Chen, 2020). Visual plasticity has also been described in adult animals, where changes in responses to familiar vs. novel visual scenes have been observed (Cooke et al., 2015). Beyond the visual system, the mammalian hippocampus has been a favorite site for studies of neuronal plasticity, with Hebbian processes like long-term potentiation, long-term depression and spike-timing dependent plasticity representing well-described mechanisms for altering information flow based on input patterns (Bliss and Lomo, 1973; Bear and Malenka, 1994; Bear and Abraham, 1996; Abbott and Nelson, 2000; Dan and Poo, 2004; Whitlock et al., 2006; Kessels and Malinow, 2009; Nicoll, 2017; Brzosko et al., 2019; Harris, 2020; Magee and Grienberger, 2020). Such studies have highlighted the general concept that specific neuronal populations display unique forms of plasticity mechanisms, with the underlying pathways often changing or disappearing over the life of any individual neuron.

Synaptic competition is a widespread form of plasticity that leads to robust synaptic inputs being strengthened and weaker inputs undergoing elimination, occurring in both the CNS and peripheral nervous system (PNS) of vertebrates (Lichtman and Colman, 2000; Holtmaat and Caroni, 2016). Synaptic competition at developing NMJs has become a widely studied model for axonal and synaptic pruning (Sanes and Lichtman, 1999; Walsh and Lichtman, 2003). At vertebrate NMJs, muscles are initially innervated by multiple motoneurons that compete through an activity-dependent process until each muscle fiber is innervated by a single neuron (Walsh and Lichtman, 2003). Although invertebrate brains are smaller and have fewer neurons, both invertebrate and vertebrate nervous systems can alter their functional connectivity in response to changes in neuronal firing patterns or behavioral experiences (Davis, 2006; Mayford et al., 2012; Giurfa, 2013; Cognigni et al., 2018; Ghelani and Sigrist, 2018). Despite reductions in overall neuron number, invertebrates are capable of concept learning, pessimistic biases, fear conditioning and attention-like processes (Giurfa, 2013). Indeed, numerous single gene mutations have been found that disrupt short or long-term memory in Drosophila, many of which are also required for mammalian learning (Davis, 2005; Androschuk et al., 2015; Crocker et al., 2016). Invertebrate neurons also show structural plasticity associated with changes in activity. For example, unilateral deafferentation, destruction or interruption of incoming connections of olfactory receptor neurons (ORNs) in the Drosophila CNS results in a significant increase of axon density of contralateral projections from the intact antenna (Berdnik et al., 2006; Golovin et al., 2019), indicating ORNs axons are capable of enhanced axonal and synaptic growth. Exposure of Drosophila to long-term odors results in olfactory adaptation and a decrease in volume of specific glomeruli, linking structural brain plasticity to learning in this model (Devaud et al., 2001). Similarly, ablation of specific motoneurons can result in axonal sprouting of neighboring neurons at the Drosophila NMJ that form new connections onto de-innervated muscles (Chang and Keshishian, 1996). Given synaptic plasticity in response to altered neuronal activity or behavioral experiences is a widespread phenomenon, many of the associated molecular and cellular mechanisms are likely to have emerged early in brain evolution (Davis, 2006; Mayford et al., 2012; Giurfa, 2013; Cognigni et al., 2018; Ghelani and Sigrist, 2018). As such, defining how distinct populations of invertebrate neurons display unique synaptic properties and plasticity mechanisms is likely to provide broader insights into how individual neuronal subtypes alter their properties during development or in response to changes in input.

Neuromuscular junctions are not classically considered highly plastic synapses, but mutants in many of the genes required for learning and memory in the Drosophila brain also show defects in synaptic function or morphology at the NMJ (Davis et al., 1998; Pan et al., 2004; Guan et al., 2011; Menon et al., 2013; Harris and Littleton, 2015; Phan et al., 2019; Bai and Suzuki, 2020). As a glutamatergic synapse, the Drosophila NMJ has become a popular model for characterizing synaptic plasticity mechanisms that may be unique to neurons using this neurotransmitter system (Harris and Littleton, 2015). The robust expansion of the NMJ over ∼6 days of larval development has made it particularly attractive for studying structural plasticity and synaptic growth regulation. In addition, this connection displays robust forms of acute and chronic homeostatic plasticity when synaptic transmission is perturbed (Frank, 2014; Davis and Müller, 2015; Cunningham and Littleton, 2019a,b; Frank et al., 2020). Most of the previous studies in these areas failed to examine differences in how tonic and phasic motoneurons express plasticity, as gaining access to synaptic function for each subtype in co-innervated muscles was difficult. Recently, distinct GAL4 drivers for subsets of Ib and Is motoneurons have been identified that allow manipulation of neuronal activity or gene function specifically in one of the two subtypes (Figures 1C–F). These drivers allow labeling of a few Ib motoneurons, including the pair that innervates muscle 1 in each abdominal segment of the animal (Figures 1C,D). In addition, several GAL4 lines allow labeling of the Is motoneuron population (Figures 1E,F), generating renewed interest in defining how these motoneurons differ in their properties and plasticity mechanisms (Newman et al., 2017; Li et al., 2018; Genç and Davis, 2019; Pérez-Moreno and O’Kane, 2019; Aponte-Santiago et al., 2020; Karunanithi et al., 2020b; Wang et al., 2020). In addition, several studies have begun to examine if tonic and phasic motoneurons display competitive or cooperative interactions during muscle innervation, or show compensatory changes when one of the motoneuron inputs is removed or altered.

The earliest differences between Drosophila tonic and phasic motoneurons were described for short-term plasticity (Kurdyak et al., 1994). Presumably as a consequence of the differences in *P*_*r*_ and RRP size, tonic Ib synapses typically display short-term facilitation while phasic Is synapses undergo short-term depression during high frequency trains (Lnenicka and Keshishian, 2000; Peled and Isacoff, 2011; Lu et al., 2016; Newman et al., 2017). Ib terminals also have a larger number of weak and silent synapses that can be recruited during short-term plasticity, while Is boutons have fewer silent AZs and their higher *P*_*r*_ sites depress (Newman et al., 2017; Akbergenova et al., 2018). These differences make Ib terminals more resistant to depression during long-term neuronal stimulation. Similar differences in short-term plasticity have been described in crayfish (Lnenicka, 2020). Crayfish NMJs also undergo long-term forms of facilitation where enhanced release can last for several hours following high frequency stimulation paradigms (Wojtowicz et al., 1994). This long-lasting effect is associated with structural changes that result in more AZs with multiple dense bodies that provide additional sites for SV release. There is also evidence that Drosophila AZs undergo rapid synaptic remodeling during homeostatic plasticity with increased accumulation of numerous AZ proteins that enhance presynaptic release (Weyhersmüller et al., 2011; Böhme et al., 2019; Goel et al., 2019; Gratz et al., 2019). There are disagreements on how much new material can be rapidly added to AZs during homeostatic plasticity vs. rearrangements of existing material into a more compact state that supports enhanced release (Mrestani et al., 2019). Further studies will be required to define the extent of AZ protein rearrangement vs. new protein addition during short-term plasticity at the NMJ.

Recent work investigating synaptic differences in tonic vs. phasic motoneurons has largely focused on homeostatic plasticity. In this form of plasticity, synaptic activity is maintained within specific ranges through homeostatic mechanisms that set a desired baseline level of output in a variety of neuronal types from invertebrates to humans (Pozo and Goda, 2010; Turrigiano, 2012; Davis and Müller, 2015). Perturbations that destabilize neurotransmitter release are offset by changes to post-synaptic receptors (synaptic scaling) and/or changes to presynaptic neurotransmission that maintain levels of output within a set range. The best characterized forms of homeostatic plasticity at Drosophila NMJs occur either through a chronic pathway following genetic disruption of post-synaptic glutamate receptor function or via acute mechanisms following pharmaceutical blockage of glutamate receptors (Petersen et al., 1997; Davis et al., 1998; Frank et al., 2006; Younger et al., 2013; Wang et al., 2014; Kiragasi et al., 2017). This process is known as presynaptic homeostatic potentiation (PHP) and requires retrograde signals from the muscle to trigger a compensatory enhancement in the number of SVs released that brings neurotransmission back to baseline levels (Frank, 2014). In addition to PHP, presynaptic homeostatic depression (PHD) has also been identified at Drosophila NMJs. PHD results in a reduction in released SVs (quantal content) following overexpression of the vesicular glutamate transporter (vGlut) that increases SV size and the amount of neurotransmitter released from individual SVs (Daniels et al., 2004; Gaviño et al., 2015). Both PHP and PHD can be co-expressed at individual NMJs (Li et al., 2018). Several recent findings highlight differences in the ability of tonic and phasic motoneurons to express homeostatic plasticity. In particular, although Ib and Is terminals can both express PHD by reducing neurotransmitter release following overexpression of vGlut (Li et al., 2018), Ib neurons appear more responsive to expressing certain forms of PHP (Ashley and Budnik, 2017; Newman et al., 2017; Li et al., 2018; Cunningham and Littleton, 2019b; Genç and Davis, 2019).

Differences in homeostatic plasticity mechanisms in Ib and Is neurons were initially observed using optical quantal imaging in GluRIIA mutants. Loss of this glutamate receptor subtype causes a decrease in post-synaptic current and mini amplitude at both Ib and Is boutons, but only Ib boutons showed PHP and elevated AZ *P*_*r*_ to drive compensation for the reduction in quantal size (Newman et al., 2017). The difference in Ib vs. Is homeostatic induction was mapped to reduced post-synaptic CAMKII activity that specifically occurred at PSDs apposed to Ib AZs. Given the higher levels of GluRIIA at PSDs of Ib AZs (Aponte-Santiago et al., 2020), there may be unique signaling mechanisms engaged at these terminals or the overall reduction in current flow in GluRIIA mutants could be more dramatic than that at Is PSDs. The muscle is likely to distinguish Ib from Is inputs by reacting to the differential Ca^2+^ influx around post-synaptic Ib synapses. Given Ib inputs have extended periods of activity during locomotion, enhanced Ca^2+^ accumulation occurs more within their terminals vs. those of Is neurons. CAMKII could then differentially respond to the distinct Ca^2+^ accumulations occurring within the PSD of the two inputs (Stratton et al., 2013). Consistent with enhanced PHP occurring at Ib terminals, the previously described AZ remodeling with enhanced accumulation of BRP and presynaptic Ca^2+^ channels that occurs during PHP was only observed at Ib terminals (Li et al., 2018). These experiments led to a hypothesis that PHP was only expressed at tonic synapses.

In contrast to the previous studies, more recent work suggests Is neurons are also likely to engage in PHP, but in distinct ways from their Ib counterparts (Cunningham and Littleton, 2019b; Genç and Davis, 2019). Using GAL4 drivers specific for each subclass, optogenetic approaches revealed both tonic and phasic motoneurons participated in acute PHP following incubation of larvae with the glutamate receptor antagonist philanthotoxin (PhTx). Under these conditions, Is motoneurons showed more robust potentiation of quantal content in low Ca^2+^ conditions where they normally have enhanced release due to their higher *P*_*r*_. In contrast to acute PHP, Ib motoneurons expressed more robust chronic PHP at lower Ca^2+^ levels in GluRIIA mutants. Chronic PHP expression was found to be sensitive to the slow Ca^2+^ chelator EGTA, suggesting changes in the coupling distance of SVs to release sites is likely changing and may differentially impact Ib vs. Is AZs (Genç and Davis, 2019). Overall, these findings suggest acute forms of homeostatic plasticity in low extracellular [Ca^2+^] are more robustly expressed at phasic terminals, while chronic homeostatic plasticity is expressed at greater levels from tonic synapses. The differential sensitivity of acute vs. chronic plasticity to EGTA argues this transition requires a change in the spacing or morphology of release sites that allows loosely coupled SVs to be exocytosed specifically at tonic Ib synapses.

**FIGURE 3:**
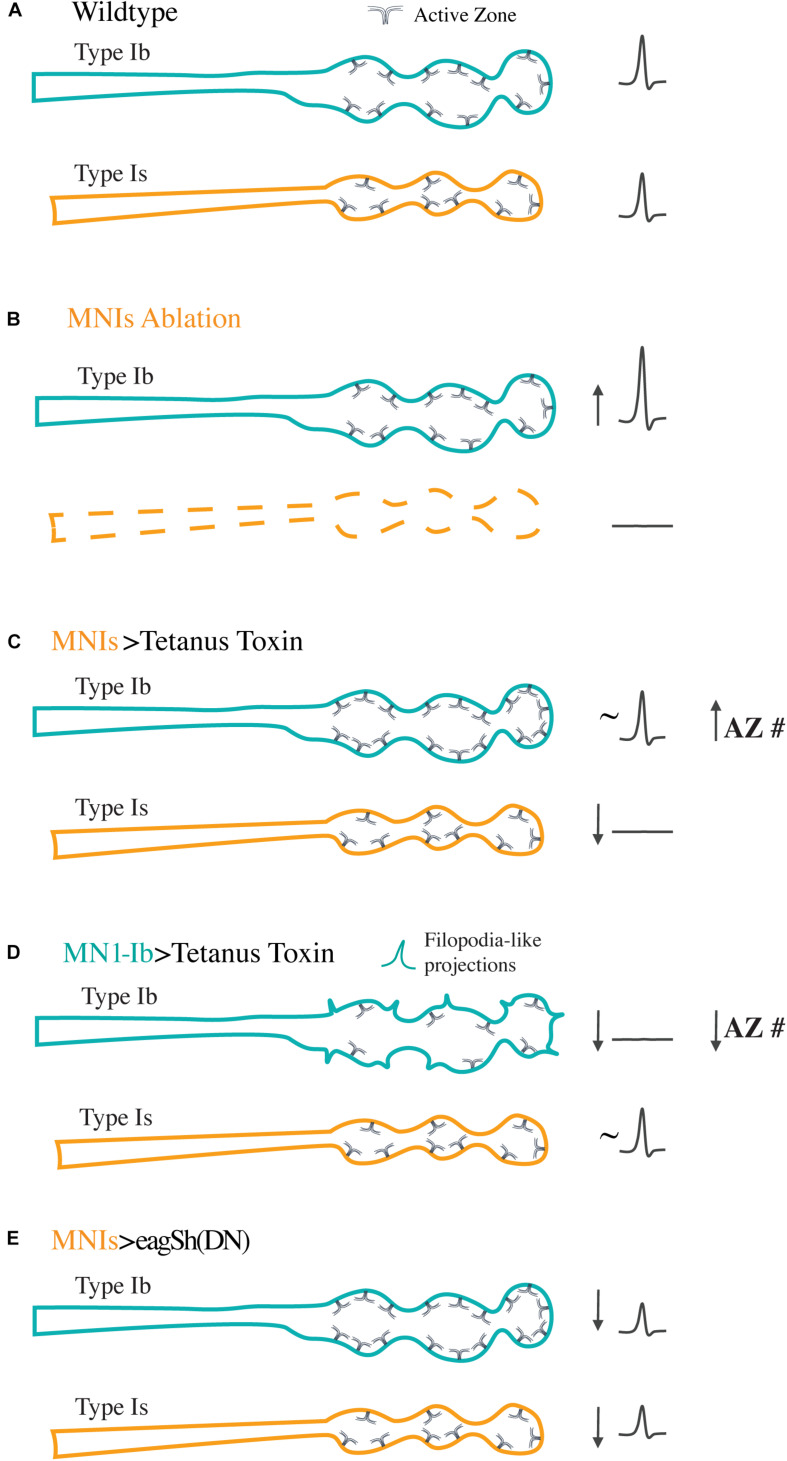
Synaptic plasticity following manipulation of the activity or presence of tonic Ib or phasic Is motoneurons. Diagrams represent the responses of Ib (teal) and Is (orange) motoneurons co-innervating the same muscle following the indicated manipulations. **(A)** For control Ib and Is terminals at muscle 1, the two inputs provide similar synaptic drive to the muscle as represented by the similar evoked excitatory junctional potentials (eEJPs) recorded from the muscle upon stimulation of either input (right). Ib NMJs contain more AZs than their Is counterparts, with an overall lower *P*_*r*_ per AZ. **(B)** Following ablation of the Is motoneuron with the Reaper cell death gene, the Ib motoneuron compensates by increasing the amount of neurotransmitter it releases without changes to AZ number. In contrast, ablation of the Ib motoneuron does not alter the structure or function of the co-innervating Is input. **(C)** Silencing the Is motoneuron with tetanus toxin results in a compensatory structural response in the co-innervating Ib input that arises from an in increase in AZ number. No structural changes are found in the silenced Is. **(D)** Silencing of the tonic Ib neuron results in reduced output, decreased AZ number, and increased filopodia-like projections in Ib with no compensatory response in the co-innervating Is input. **(E)** Increasing activity of the Is motoneuron by overexpressing dominant negative K^+^ channels to elevate overall firing rates results in uniform downscaling of evoked release in both the Ib and Is inputs as a compensation mechanism.

The identification of GAL4 drivers that specifically label tonic and phasic motoneurons (Figures 1C–F) has also allowed studies of how each class responds to manipulations of their co-innervating partner. Studies from our group identified GAL4 drivers for Ib and Is motoneurons innervating muscle 1 and used these lines to probe how alterations in the activity or presence of each motoneuron could change the properties of the other class of inputs (Aponte-Santiago et al., 2020). At this muscle, synaptic output driven by the two neuronal subclasses is normally matched, with each contributing similar levels of synaptic drive for muscle contraction. At the structural level, no evidence for either competitive or cooperating signals were found to influence synaptic growth of the two inputs during development. This contrasts with the highly competitive motoneuron elimination process that occurs at mammalian NMJs (Walsh and Lichtman, 2003; Tapia et al., 2012). Although there was no evidence of competition between the two inputs during innervation of the same target, compensation responses were observed in Ib motoneurons when the co-innervating Is input was ablated or silenced (Figures 3A–C). Complete loss of the Is input resulted in enhanced synaptic output and more neurotransmitter release from the Ib motoneuron without a corresponding change in synaptic bouton or AZ number. In contrast, silencing of the Is input with genetically expressed tetanus toxin to block SV fusion resulted in structural changes with more AZs forming in the co-innervating Ib input. In contrast, silencing of Ib motoneurons did not result in any observable compensatory changes in Is synaptic structure or function (Figure 3D), suggesting tonic Ib inputs are the primary subclass of glutamatergic motoneurons responding to reduced input of their co-innervating partner. Silencing of neuronal activity during development has also been shown to induce ectopic NMJs formed by type II neuromodulatory neurons (Keshishian et al., 1994; Jarecki and Keshishian, 1995; White et al., 2001; Lnenicka et al., 2003; Mosca et al., 2005; Carrillo et al., 2010; Vonhoff and Keshishian, 2017), indicating both type Ib and type II neuronal subclasses display a capacity for structural plasticity when muscle input is reduced.

More recent work in this area has also shown that Ib plasticity extends beyond the muscle 1 inputs previously characterized. Carrillo et al. (2010) performed a similar analysis using Is manipulations and examined a larger subset of muscle fibers (Wang et al., 2020). They observed compensatory increases in synapse number and neurotransmitter release in Ib motoneurons that mirrored the pre-existing strength of the co-innervating Is input. Manipulation of Is in muscles where the input was normally strongest elicited the largest compensating responses from the co-innervating Ib input. For muscles with normally weak Is input, no compensation in the co-innervating Ib input was observed. Interestingly, complete elimination of the Is input at specific muscles using mutations in the Dip-α synaptic targeting cell surface receptor prevented robust Ib plasticity responses, suggesting co-innervation is also required for the magnitude of Ib compensation. We also observed differences in Ib compensation at muscle 1 depending on whether Is was present or silenced. Although Ib increased synaptic output following loss of the Is, the increase in AZ number in Ib motoneurons following expression of tetanus toxin in Is neurons was not observed when Is was ablated (Aponte-Santiago et al., 2020). These differences in plasticity responses indicate structural changes require the physical presence of a non-functional Is NMJ on the muscle. These data also suggest muscles are able to distinguish when the Is motoneuron is absent, vs. present and non-functional, resulting in distinct responses that ultimately trigger structural or functional changes at Ib synapses.

Tonic and phasic neurons also show distinct responses to silencing of their own activity. When activity was reduced with tetanus toxin in Ib motoneurons (Figure 3D), there were striking alterations in their structure that included increased presynaptic filopodia and reduced AZ number and post-synaptic SSR development (Aponte-Santiago et al., 2020). These features are reminiscent of immature synapses, suggesting activity at Ib synapses plays an important role in their subsequent maturation. This effect was only observed at Ib terminals, indicating Is neurons that are silenced interact with or respond to signals from the muscle in a distinct way that doesn’t appear to alter their structure. Although the underlying logic for these differences in plasticity responses is unknown, one can speculate that plasticity of tonic motoneurons may be more relevant in Drosophila since each muscle is innervated by only a single Ib motoneuron that is the primary driver for contraction (Newman et al., 2017). Even though phasic Is neurons display less plasticity at muscle 1, it is possible smaller plastic changes are occurring in this subtype but are not expressed in a target-specific manner. Given individual Is motoneurons innervate many muscles, unlike Ib neurons that target a single muscle, changes in the Is neuron could be distributed over more synapses onto a larger muscle population, resulting in little effect at any single target. Alternatively, Is motoneurons may be less sensitive to any putative muscle-derived retrograde signals that trigger plasticity in response to reduced muscle input. Finally, there could also be compartmentalization in the release of post-synaptic retrograde signals from the muscle that result in activation of only the Ib terminals.

Although we did not find changes in the morphology or output of Is or Ib motoneurons following moderate increases in their individual spiking activity, morphological changes have been observed with pan-neuronal increases in activity (Budnik et al., 1990; Jia et al., 1993; Rieckhof et al., 2003; Sigrist et al., 2003; Guan et al., 2005; Mosca et al., 2005; Yoshihara et al., 2005; Huntwork and Littleton, 2007; Ataman et al., 2008; Barber et al., 2009; Piccioli and Littleton, 2014; Cho et al., 2015). These studies indicate increases in evoked or spontaneous release from motoneurons can drive the formation of extra synaptic boutons and AZs during larval development. In addition, more robust changes induced by overexpression of dominant-negative K^+^ channels specifically in Is motoneurons has been shown to alter the output of both the Is and co-innervating Ib input (Karunanithi et al., 2020b). In these experiments, the activity of the Is was driven to a highly active spiking state during late larval development or more acutely within specific temporal windows. Under both conditions, the Is motoneuron and the unaffected Ib neuron innervating the same muscle responded by downscaling their evoked synaptic transmission without alterations to the number of synapses they formed with the muscle (Figure 3E). Although synaptic transmission was decreased at both synaptic terminals to compensate for the enhanced activity of the Is motoneuron, the underlying mechanisms were specific to each neuronal class. In the case of Ib, there was a decrease in quantal content and the number of SVs released in response to stimulation that resulted in a reduced P_r_. Although Is neurons showed a decrease in quantal content as well, they displayed an increase in quantal size reflected in larger mini amplitudes that was linked to changes in expression of the vGlut transporter in Is neurons. This effect did not occur in Ib terminals, suggesting distinct plasticity mechanisms can be engaged in tonic vs. phasic motoneurons following either increases or decreases in their activity.

Motoneuron innervation is normally hard-wired in Drosophila larvae, with individual muscles allowing synaptic innervation from only a single motoneuron of each subclass. However, the examples described above indicate plasticity can occur when the activity or presence of co-innervating inputs are altered. Additional examples of motoneuron plasticity are also observed when a post-synaptic target is eliminated or the cell-surface proteome of the motoneuron is altered. Ectopic innervation of muscles has been observed following muscle loss induced by laser ablation or genetic mutation, with the affected Ib motoneuron inappropriately synapsing onto nearby muscles (Sink and Whitington, 1991a; Keshishian et al., 1994; Chang and Keshishian, 1996). Similarly, laser ablation of motoneurons results in axonal spouting from nearby unaffected motoneurons that subsequently target de-innervated muscles (Chang and Keshishian, 1996). Mis-expression of synaptic cell surface proteins on the motoneurons themselves can also change Ib and Is innervation, resulting in synapses on inappropriate muscle targets (Lin and Goodman, 1994; Kose et al., 1997; Shishido et al., 1998; Ashley et al., 2019). These observations indicate the Drosophila motor system differs from vertebrate NMJs, with motoneurons normally displaying an autonomous role in target selection without competition from other motoneurons of the same class. An interesting scenario will be to examine within class neuronal competition using genetic manipulations that result in ectopic muscle innervation by two neurons of the same class, providing a more similar situation to mammalian NMJs. Indeed, poly-innervation of dorsal larval muscles by multiple Ib motoneurons can be triggered by manipulating transcription factors specifying dorsal Ib cell fate (Meng et al., 2020, 2019). These manipulations induce poly-innervation from an expanded Ib lineage. Optical imaging of synaptic activity has confirmed that duplicated neurons release neurotransmitters onto hyper-innervated muscles. It will be interesting to determine if these multiple Ib inputs display competition for synaptic drive as observed at mammalian NMJs. Although manipulations that induce phasic Is co-innervation have not yet been identified, such studies would provide an informative test for intraclass phasic motoneuron competition as well.

## Conclusion and Future Directions

An important question in tonic and phasic motoneuron biology moving forward is what distinct mechanisms underlie the unique biophysical, synaptic structure, release properties and plasticity mechanisms that distinguish the two neuronal subgroups. Differences in Is and Ib properties are ultimately controlled by expression of specific transcription factors expressed in each lineage. Indeed, manipulating key transcription factors required for motoneuron development can alter synaptic target choice during larval development (Meng et al., 2020). Similar manipulations would be expected to regulate other intrinsic neuronal properties as well. Identifying the cell surface proteome from each subclass would also help define mechanisms for why Ib motoneurons innervate single muscles while Is motoneurons innervate multiple targets. As a first step toward these goals, the specific transcriptomes of tonic and phasic motoneurons are starting to be examined using RNA sequencing approaches (Genç and Davis, 2019). As this work progresses, characterizing the unique gene expression signatures and their specific roles in tonic and phasic neuronal properties should generate exciting clues into how neuronal diversity mechanisms arise via distinct transcriptional programs. Similarly, defining alternative splicing differences between tonic and phasic motoneurons may reveal unique splice variants specific to one subtype or the other. The discovery of GAL4 drivers uniquely expressed in tonic or phasic motoneurons now allows RNAi and CRISPR-based gene disruption studies to be targeted specifically to each subclass. In addition, overexpression approaches can be used with these drivers to identify genes capable of switching tonic vs. phasic properties when differentially expressed between the two neuronal classes.

Future studies should also be able to identify the mechanisms that allow structural and functional plasticity in tonic Ib motoneurons following manipulations of the co-innervating phasic Is input. Defining why phasic Is neurons fail to respond as robustly to these manipulations will also be of interest. Key differences in molecular signaling and synaptic receptors expressed by the two subtypes is likely to be important for their differential plasticity mechanisms. Indeed, the classic BMP synaptic growth regulator glass bottom boat (Gbb) appears to have both shared and distinct roles in the two neuronal populations (Aponte-Santiago et al., 2020). In addition, whether homeostatic mechanisms triggered in response to acute or chronic reduction in glutamate receptor function are similarly employed for plasticity triggered by the absence or functional silencing of motoneuron inputs are important questions. Many molecular components have already been characterized for their role in homeostatic plasticity (Davis, 2006, 2013; Bergquist et al., 2010; Müller et al., 2011, 2012; Müller and Davis, 2012; Younger et al., 2013; Frank, 2014; Wang et al., 2014, 2016; Davis and Müller, 2015; Gaviño et al., 2015; Kiragasi et al., 2017; Li et al., 2018; Ortega et al., 2018; Böhme et al., 2019; Goel et al., 2019; Gratz et al., 2019; Frank et al., 2020). These include pathways that result in functional increases in presynaptic output due to increased SV pools, increased presynaptic Ca^2+^ influx, or enhanced membrane excitability. Numerous pathways that control structural plasticity and regulation of AZ number at Drosophila NMJs have also been identified, including Neurexin/Neuroligin, Teneurins, neurotrophins, Synaptotagmin 4-mediated retrograde signaling, BMPs, Wingless, and regulated proteolysis (Wan et al., 2000; Aberle et al., 2002; Marqués et al., 2002; DiAntonio and Hicke, 2004; Yoshihara et al., 2005; Ataman et al., 2006, 2008; Collins et al., 2006; Collins and DiAntonio, 2007; Li et al., 2007; Johnson et al., 2009; Wairkar et al., 2009; Banovic et al., 2010; Sun et al., 2011; Miller et al., 2012; Mosca et al., 2012; Owald et al., 2012; Berke et al., 2013; Korkut et al., 2013; Piccioli and Littleton, 2014; Harris and Littleton, 2015; Muhammad et al., 2015; Harris et al., 2016; Banerjee et al., 2017; Ulian-Benitez et al., 2017). Defining if and how these well-known molecular pathways for synaptic growth and function are uniquely employed in tonic vs. phasic motoneurons should complement RNA profiling approaches and help decipher how differences in synaptic structure and function arise.

In addition to changes in the transcriptome of tonic and phasic motoneurons, post-translational modifications to ion channels and other synaptic proteins may alter their location or function differentially within the two neuronal classes. Altered phosphorylation of key components of the SV fusion machinery would be an interesting target for the dynamic differences in Ca^2+^ sensitivity of release between the two classes. It remains to be seen what parallels at the molecular level are present between invertebrate tonic and phasic neurons and vertebrate neurons with similar properties. Once the underlying mechanisms are identified in a genetically approachable system like Drosophila, it will provide a robust set of hypotheses that can be tested in mammalian models. In conclusion, further dissection of the pathways that generate the unique properties of invertebrate tonic and phasic neurons will no doubt continue to provide important insights into neuronal diversity mechanisms that contribute to biophysical properties, synaptic target choice, synapse structure and function, and differential synaptic plasticity pathways.

## Author Contributions

All authors listed have made a substantial, direct and intellectual contribution to the work, and approved it for publication.

## Conflict of Interest

The authors declare that the research was conducted in the absence of any commercial or financial relationships that could be construed as a potential conflict of interest.
